# Validating the Time and Change test to screen for dementia in elderly Koreans

**DOI:** 10.1186/1471-2458-4-52

**Published:** 2004-11-04

**Authors:** Jung-Ae Rhee, Eun-Kyung Chung, Min-Ho Shin

**Affiliations:** 1 Department of Preventive Medicine, Chonnam National University Medical School, Hak-1-dong, Dong-gu, Gwangju 501–190, South Korea; 2 Department of Preventive Medicine, Seonam University College of Medicine, 120–1, Mareuk-dong, Seo-gu, Gwangju, South Korea

## Abstract

**Background:**

We assessed the applicability of the T&C test as an accurate and convenient means to screen for dementia in primary care and community settings.

**Methods:**

The study group comprised 59 patients and 405 community participants, all of who were aged 65 years and over. The time component of the T&C test evaluated the ability of a subject to comprehend clock hands that indicated a time of 11:10, while the change component of the T&C test evaluated the ability of a subject to make 1,000 Won from a group of coins with smaller denominations (one 500, seven 100, and seven 50 Won coins).

**Results:**

The T&C test had a sensitivity and specificity of 73.0 and 90.9%, respectively, and positive and negative predictive values of 93.1, and 66.7%, respectively. The test-retest and interobserver agreement rates were both 95% (κ = 0.91) (time interval, 24 hours). The association between the T&C test and K-MMSE test was modest, while significant (r = 0.422, p < 0.001). The T&C test scores were not influenced by educational status.

**Conclusions:**

We conclude that the T&C test is useful as supplemental testing of important domains (e.g., calculation, conceptualization, visuospatial) to traditional measures such as the MMSE. However, because T&C test is simple, rapid, and easy to use, it can be applied conveniently to elderly subjects by non-specialist personnel who receive training.

## Background

Dementia, an acquired persistent impairment of cognitive functioning is an increasingly common problem in Korea, and is associated with increased morbidity and mortality, functional loss, caregiver burden, and institutionalization [1]. Nevertheless, most patients with dementia are not detected by family members or clinicians at an early stage, even though such patients have memory problems [2,3].

Screening for dementia has been recommended to increase detection of dementia. The Mini-Mental State Examination (MMSE) is a brief screening test that quantitatively assesses the cognitive status of elderly people [4,5]. Although it has shown good validity, the MMSE has been found to be influenced largely by racial differences and educational status [6-10]. Korean elderly show very low levels of educational background, and it is necessary to develop appropriate cognitive assessment method that is not influenced by education. The Time and Change (T&C) test was developed by Inouye et al. [11] to assess (i) how well a patient understands time and (ii) the ability of the patient to calculate using money. Several studies have found that the T&C test allows direct assessment of two important activities of daily living and supplements the testing of calculation, conceptualization, and visuospatial cognitive domains irrespective of race and education [11-13].

We have therefore introduced a T&C test, and assessed the applicability of the T&C test as a convenient and accurate means to detect early stage dementia in primary care and community settings.

## Methods

### Subjects

One study group initially comprised 60 participants who either visited or were admitted to a hospital that was located in an urban area, namely Gwangju city (referred to forthwith as area A) between November and December 2001. Of these 60 participants, 37 were diagnosed with Alzheimer's disease or vascular dementia, 11 had mental illness such as schizophrenia or depression, 7 had organic brain disease such as cerebral apoplexy or Parkinson's disease, and 5 were alcoholics.

Another study group initially comprised 412 participants who were recruited from all residents of Jangseong-county, Jeonnam province, South Korea, aged 65 and over in 2002(referred to forthwith as area B). The area consists of 11 towns and had an estimated population 54,528 of whom about 16.5% were aged 65 and over. The subjects were selected from each stratum (town) using cluster sampling. All subjects in whom vision or hearing was impaired were excluded from the study. We selected 59 (of 60) and 405 (of 412) participants from area A and B, respectively. All participants gave informed consent, and when participants with dementia cannot provide informed consent, caregivers were asked to provide it.

### The Time and Change test

The 'time' component of the T&C test evaluated the ability of a subject to comprehend that the hands of a clock indicated 11:10. The diameter of the clock was 15 cm, and the distance between the clock and the participant was 25–35 cm (in consideration of the visual deficits of the older subjects). When the subject responded incorrectly on the first attempt, the interviewer posed the same question, i.e., a second attempt was permitted. The time (in seconds) that it took for the participant to respond correctly was recorded. A time limit of 60 s for a response was imposed.

In the 'change' component of the T&C test, the participants were required to make 1,000 Won from a group of coins of several smaller denominations, namely one 500, seven 100, and seven 50 Won coins, that were placed on a table in front of the participant. The participants were given 120 s to complete the task, and an additional 120 s was granted if the subject failed to complete the task at the first attempt. The time (in seconds) that it took for the participant to complete the task was recorded by the interviewer.

The participants who completed both of the aforementioned tasks of the T&C test successfully were determined to be negative for suspected dementia, whereas participants who failed to complete either or both of the tasks were determined to be positive for suspected dementia. The T&C test was performed prior to the other cognitive tests by an interviewer who was unaware of the results of the other tests.

### Measurement

In the participants from area A, the T&C test was conducted by a nurse, while a physician who were blinded to the result of the T&C test performed a clinical examination and neuropsychiatric inventory. The diagnostic criteria for dementia were based on those of the Diagnostic and Statistical Manual of Mental Disorders (Fourth Edition) [14]. To detect the causative diseases of dementia, the following were carried out: general blood and chemical tests (including VDRL, Vitamin B12, T3, T4, TSH); a chest radiograph; an electrocardiogram; a brain computerized tomography scan; and psychometric tests including the K-MMSE [15]. Alzheimer's dementia was diagnosed based on the guidelines of the National Institute of Neurological and Communicative Disorders and Stroke and Alzheimer's disease and Related Disorders Association (NINCDS-ADRDA) [16]. Vascular dementia was diagnosed based on the guidelines of the National Institute of Neurological Disorders and Stroke and the Association Internationale pour la Recherche et l'Enseignement en Neurosciences (NINDS-AIREN) [17]. The CDR developed by Hughes et al. [18] was used to determine the progression of dementia.

The participants from area B were interviewed by interviewers who had undergone sufficient training to be able to conduct the K-MMSE and T&C test.

All participants were interviewed for data on social and demographic factors such as address, age, sex, educational status, and the number of family members living together.

### Assessment of the validity of the T&C test

To assess the validity of the T&C test as a method to screen for dementia in the participants from area A, we compared the results of the test to a reference standard (diagnosed by a physician), and evaluated sensitivity, specificity, and positive and negative predictive values. To assess the test-retest reliability, the one type of interviewer (specifically, physicians) conducted the same test twice at an interval of 24 h (n = 22 participants). To assess the interobserver reliability, two different types of interviewer (specifically, physicians and psychometrists) conducted the same test, at an interval of 24 h (n = 22 participants).

To assess the applicability of the T&C test as a method to screen for dementia in the participants from area B, the association between the T&C and K-MMSE test scores was evaluated.

### Statistical analysis

The sensitivity, specificity, and positive and negative predictive values and 95% Confidence interval of the T&C test were analyzed. After classifying the participants from area B into a group in which dementia was suspected, and another group in which dementia was not suspected based on the results from the T&C test, a Student's t-test was used to compare the total K-MMSE score with the scores for each of the components of the K-MMSE test, and Spearman's rank correlation was used to analyze the correlation between the T&C and K-MMSE test scores. SPSS for Windows (version 10.0; SPSS Inc., Chicago, IL, USA) and Stata Software 6.0 (Stata Corporation, College Station, Texas) were used to analyze data and statistics.

## Results

### Characteristics of subjects

For the participants from area A, the average age was 73.2 ± 7.9 years, 55.9% of the group was female, the average number of years of education was 4.2 ± 5.4, and 62.7% lived alone. For the participants from area B, the average age was 73.1 ± 6.1 years, 58.5% of the group was female, the average number of years of education was 3.1 ± 4.0, and 29.0% lived alone (Table 1).

**Table 1:** Characteristics of the study groups.

Characteristic	Hospital* (n = 59)	Community^† ^(n = 405)
Age: years, mean ± SD	73.2 ± 7.9	73.2 ±
(range)	(58–90)	(65–97)
Gender: female, n (%)	33 (55.9)	238 (58.5)
Education: years, mean ± SD	4.2 ± 5.4	3.1 ± 4.0
(range)	(0–16)	(0–16)
Living alone: n (%)	32 (62.7)	118 (29.0)

SD = standard deviation.

*Referred to in main text as area A (see Methods).

^†^Referred to in main text as area B (see Methods).

### Validity of the T&C Test

Table 2 shows the results of an analysis of the validity of the T&C test. In a comparison of the T&C test and a diagnosis of dementia by physicians (which served as a reference), 27 of 37 patients that had been diagnosed as demented were classified as positive for dementia according to the T&C test, and the sensitivity was 73.0%. Of the subjects who were diagnosed as not demented, 20 of 22 were classified as negative according to the T&C test, and the specificity was 90.9%. The positive predictive value that indicated the probability of dementia, as determined by a positive classification according to the T&C test, was 93.1%, while the negative predictive value that indicated the probability of not being demented, as determined by a negative classification according to the T&C test, was 66.7%.

**Table 2:** Concurrent validity of the Time and Change (T&C) test in a sample of elderly hospital patients.

		Reference standard*		Total
			
		Dementia	No dementia	
T&C	+	27	2	29
assessment	-	10	20	30
	Total	37	22	59
Sensitivity = 27/37 (73.0%) (CI = 61.6–84.3%)				
Specificity = 20/22 (90.9%) (CI = 83.6–98.2%)				
Positive predictive value = 27/29 (93.1%) (CI = 86.6–99.6%)				
Negative predictive value = 20/30 (66.7%) (CI = 54.6–78.7%)				
Accuracy = 47/59 (79.7%)				

CI = 95% confidence interval for sensitivity and specificity values.

*Diagnosis of dementia made by a clinician.

+, positive for dementia according to T&C test.

-, negative for dementia according to T&C test.

### Reliability of the T&C test

The rate of agreement of the test-rest & interobserver variability was analyzed to assess the reliability of the T&C test. The rate of agreement of both the test-retest and interobserver was 95% (κ = 0.91) (time interval, 24 hours).

### Comparison of the T&C and K-MMSE test

There was a significant difference between the total K-MMSE score and the scores for each of the components of the K-MMSE test between participants that were classified as positive and those that were classified as negative for dementia according to the T&C test (Table 3). The association between the T&C test and K-MMSE test revealed modest, while significant (r = 0.422, p < 0.001) (Table 4).

**Table 3:** Korean Mini-Mental State Examination (K-MMSE) scores according to T&C test performance in a sample of elderly community population.

			K-MMSE components				
	K-MMSE score (overall) (n = 30)		Orientation (n = 10)	Registration (n = 3)	Concentration & Calculation (n = 5)	Recall (n = 3)	Language & Diagram (n = 9)
T&C test*	+^† ^(n = 65)	14.8 ± 5.8	6.1 ± 2.8	2.1 ± 1.1	0.5 ± 1.4	0.8 ± 1.1	5.4 ± 1.7
	- (n = 340)	22.4 ± 5.0	8.9 ± 1.8	2.8 ± 0.6	2.2 ± 1.9	1.6 ± 1.1	7.0 ± 1.6
Time test*	+^‡ ^(n = 53)	14.3 ± 5.3	6.0 ± 2.6	2.0 ± 1.1	0.4 ± 1.2	0.7 ± 1.1	5.2 ± 1.6
	- (n = 352)	22.3 ± 5.1	8.8 ± 1.9	2.8 ± 0.6	2.2 ± 1.9	1.6 ± 1.2	7.0 ± 1.6
Change test*	+^§ ^(n = 31)	14.6 ± 6.9	6.0 ± 3.0	2.0 ± 1.2	0.8 ± 1.7	0.5 ± 1.0	5.3 ± 2.0
	- (n = 374)	21.8 ± 5.4	8.6 ± 2.0	2.7 ± 0.6	2.0 ± 1.9	1.5 ± 1.2	6.8 ± 1.7

() : number mean ± SD.

*p < 0.01 for K-MMSE score (or a component thereof) versus T&C test score (Student's t-test).

+, positive for dementia according to T&C test.

-, negative for dementia according to T&C test.

^†^Incorrect response for either or both the time and change task of the T&C test (see Methods for details).

^‡^Incorrect response for the time task.

^§^Incorrect response for the change task.

**Table 4:** Convergent validity of the T&C test in a sample of elderly community population.

	Correlation coefficient (r)*
K-MMSE (overall score)	0.422
K-MMSE component scores:	
Orientation	0.431
Registration	0.331
Concentration & Calculation	0.358
Recall	0.258
Language & Diagram	0.314

*p < 0.001 for all values (Spearman's rank correlation analysis).

### Association between the T&C test and educational status

The results of a logistic regression in which the T&C test as a dichotomous and dependent variable was performed with education status adjusting for age and sex revealed no association between them. The odds ratio for T&C test associated with educational status is 0.877 (95% CI = 0.766–1.004).

### Response times in the T&C test

For the time task in the T&C test, 75.8% of the participants produced a correct response on the first attempt after 6.3 ± 6.7 s, and 45 participants (11.1%) produced a correct response on the second attempt. For the change task in the T&C test, 81.2% of the participants produced a correct response on the first attempt after 12.7 ± 14.2 s, and 43 participants (10.6%) produced a correct response on the second attempt. 34 participants(8.4%) were tested twice for both the time and change task. None of the subjects refused to respond during the tests.

## Discussion

Interracial variability in both the etiology of dementia and the accuracy of cognitive testing suggests that there is an urgent need to develop racially appropriate methods of cognitive assessment. The rate of vascular dementia due to cerebrovascular disease is much higher in Koreans than in other races; this is due to insufficient prevention, diagnosis, and treatment of hypertension, diabetes, and hyperlipidemia in Korea. Vascular dementia, unlike Alzheimer's disease, can be prevented by appropriate treatment and prevention for the risk factors of cerebrovascular diseases, can be treated to improve symptoms and inhibit progression of the disease.

In the present study, the original T&C test of Inouye et al. [11] was modified to apply specifically to Korean elderly sample, and was used to screen for dementia. We found that the sensitivity of the T&C test was 73.0%, specificity was 90.9%, and the positive and negative predictive values were 93.1% and 66.7%, respectively. The disparity between the sensitivity and the specificity might mean the insensitivity to mild stage or pre-dementia. We advocate using the T&C test sequentially with other dementia measures (with increased sensitivity).

When compared to the results of Kawamato [19], in which sensitivity and specificity was 49.1 and 95.2%, respectively; the sensitivity of the T&C test in the present study was remarkably high, while specificity was relatively low. In a study by Inouye et al. [8] in which the MMSE and modified Blessed Dementia Rating Scale (mBDRS) served as standards, sensitivity was 86%, specificity was 71%, and the negative predictive value was 97% when the subjects were hospital patients [8], while sensitivity was 63%, specificity was 96%, and the negative predictive value was 93%, for outpatients [12]. This discrepancy might be due to the characteristics of different samples or the diagnostic standards for dementia. The rate of dementia among the participants of the present study (62.7%) was higher than in the studies by Inouye and colleagues (14 and 16% for admitted and outpatients, respectively). Therefore, there are limitations when it comes to comparing the predictive values of screening tests, which would appear to be influenced by the prevalence.

In elderly patients, the assessment of cognitive function is affected by psychological factors and by the circumstances under which the tests are conducted. In our measurement of the reliability of the T&C test, the test-retest and interobserver agreement rates were both remarkably high (95% for each, κ = 0.91). In a study by Inouye et al. [8] the test-retest agreement rate was 88%, and the interobserver agreement rate was 78% when the subjects were hospital patients [8]. However in the present study test-rest was conducted in the short interval, therefore it might to be influenced by carry-over effects and learning effects.

In the present study, no association was observed between T&C test scores and educational status. This result may be explained by the fact that interpreting the hands of a clock and calculation using change are behaviors that are common to all people during daily life, irrespective of race and education. In addition, unlike the language-focused MMSE, the questions in the T&C test cannot be misinterpreted or misunderstood, and are effective for assessing calculation ability and attention. Clock test (similar to T&C test) is less likely to be confounded by educational attainment [20], and measures various facets of cognitive functioning at varying levels of difficulty [21].

This study has several important limitations. First, while the hospital patients underwent an evaluation of their medical history as well as neurological and physical examinations before a diagnosis of dementia was made, the elderly community sample were evaluated using only the K-MMSE as a reference. Second, the hospital patients would appear to be a rather unusual sample in that there was a high prevalence of psychiatric morbidity in the non-demented subjects. Third, dementia in the present study was not classified as vascular or Alzheimer's-type, nor did we consider the severity of symptoms. Finally, the T&C test has limitations in assessment of a wide range of deficits associated with dementia. However, because T&C test is simple, rapid, and easy to use, the T&C test may pose particular advantages in primary care and community settings where frequent assessment of cognitive functioning is required. The T&C test adds supplemental testing of important domains (e.g., calculation, conceptualization, visuospatial) to traditional measures such as MMSE. In addition, because the T&C test is less influenced by educational status, it may be particularly useful in populations with diverse educational and cultural backgrounds.

## Conclusions

We conclude that the T&C test is useful as a supplemental testing of important domains (e.g., calculation, conceptualization, visuospatial) to traditional measures such as the MMSE, because sensitivity of T&C test is not great and the association between the T&C test and MMSE is modest. However, because T&C test is simple, rapid, and easy to use, it can be applied conveniently to elderly subjects by non-specialist personnel who receive training. In addition, the T&C test is less influenced by educational status

## Funding

This work was supported by grants from Chonnam National University Hospital (CUHRI-U-200241).

## Competing interests

The author(s) declare that they have no competing interests.

## Authors' contributions

JAR conceived of the study, collected data and drafted the manuscript. EKC participated in its design, performed data collection, and reviewed the manuscript. MHS participated in data analysis and reviewed the manuscript. All authors read and approved the final manuscript.

## Pre-publication history

The pre-publication history for this paper can be accessed here:


